# The STING agonist, DMXAA, reduces tumor vessels and enhances mesothelioma tumor antigen presentation yet blunts cytotoxic T cell function in a murine model

**DOI:** 10.3389/fimmu.2022.969678

**Published:** 2022-11-18

**Authors:** Peter T. Graham, Anna K. Nowak, Scott M. J. Cornwall, Irma Larma, Delia J. Nelson

**Affiliations:** ^1^ School of Medicine, Curtin University, Bentley, WA, Australia; ^2^ Medical School, University of Western Australia, Nedlands, WA, Australia; ^3^ National Centre for Asbestos Related Diseases, Nedlands, WA, Australia; ^4^ Institute of Respiratory Health, Nedlands, WA, Australia; ^5^ Safe Work Laboratories, Belmont, WA, Australia; ^6^ Becton Dickinson Pty Limited, Osborne Park, WA, Australia; ^7^ Curtin Health Innovation Research Institute, Bentley, WA, Australia

**Keywords:** STING agonist, mesothelioma, tumor antigen presentation, cytotoxic T lymphocytes (CTL), IL-2, agonist anti-CD40 antibody

## Abstract

We assessed the murine Stimulator of Interferon Genes (STING) agonist, DMXAA, for anti-mesothelioma potential using the AE17-sOVA model that expresses ovalbumin (OVA) as a neo tumor antigen. Dose response experiments alongside testing different routes of administration identified a safe effective treatment regimen that induced 100% cures in mice with small or large tumors. Three doses of 25mg/kg DMXAA given intra-tumorally every 9 days induced tumor regression and long-term survival (>5 months). Re-challenge experiments showed that tumor-free mice developed protective memory. MTT and propidium-iodide assays showed that DMXAA exerted direct cytotoxic effects at doses >1mg/ml on the murine AE17 and AB1 mesothelioma cell lines. *In-vivo* studies using a CFSE-based *in-vivo* proliferation assay showed that DMXAA improved tumor-antigen presentation in tumor-draining lymph nodes, evidenced by OVA-specific OT-1 T cells undergoing more divisions. An *in-vivo* cytotoxic T lymphocyte (CTL) assay showed that DMXAA blunted the lytic quality of CTLs recognizing the dominant (SIINFEKL) and a subdominant (KVVRFDKL) OVA epitopes. DMXAA reduced tumor vessel size *in-vivo* and although the proportion of T cells infiltrating tumors reduced, the proportion of tumor-specific T cells increased. These data show careful dosing and treatment protocols reduce mesothelioma cell viability and modulate tumor vessels such that tumor-antigen specific CTLs access the tumor site. However, attempts to enhance DMXAA-induced anti-tumor responses by combination with an agonist anti-CD40 antibody or IL-2 reduced efficacy. These proof-of-concept data suggest that mesothelioma patients could benefit from treatment with a STING agonist, but combination with immunotherapy should be cautiously undertaken.

## 1 Introduction

Mesothelioma, a rare aggressive cancer that develops in mesothelial cells lining the pleura and peritoneum (1–3) is characterized by a long latency period after asbestos exposure and a poor prognosis post diagnosis on account of its relative resistance to standard treatment options such as chemotherapy and radiation. Thus, new treatment strategies are desperately required. Tumor angiogenesis is crucial for sustained development of solid tumors, and vascular-targeting strategies for mesothelioma are showing promise (4), including use of Bevacizumab (also referred to as Avastin), an anti-vascular endothelial growth factor (VEGF)A antibody combined with cisplatin and pemetrexed (4, 5). The latter was a randomized, controlled, open-label, phase 3 trial, in 448 mesothelioma patients who had not been given previous chemotherapy and did not have significant cardiovascular comorbidity. Patients were given cisplatin and pemetrexed with or without Bevacizumab; overall survival was significantly longer (by 2.7 months) in the group given Bevacizumab with manageable side effects, although some patients withdrew due to toxic effects. These results promoted us to explore the use of DMXAA as an alternative anti-angiogenic in a preclinical setting that may offer longer survival with minimal toxicity. Mesothelioma is also responsive to immunotherapy which has now entered into standard care (6–11) and there is evidence it may be responsive to Stimulator of Interferon Genes (STING) agonists (12) which is reported to target tumor blood vessels and the immune system.

Stimulator of interferon genes (STING) encoded by *TMEM173* is an endoplasmic reticulum (ER) signaling molecule that plays a key role in host defense by responding to pathogen or self-derived DNA, reviewed by (13). Activation of STING, a 379 amino acid protein, consisting of several transmembrane regions, leads to TBK1/IRF3 and NF-kappaB activation and type I interferon (IFN) production plus other pro-inflammatory cytokines (14). STING-expressing cells include endothelial and epithelial cells, and immune cells including macrophages, dendritic cells (DCs) and T cells (12, 15–18).

DMXAA (5,6-dimethylxanthenone-4-acetic acid), a flavone-acetic acid-based drug, directly binds murine STING (19, 20) has been shown to selectively destroy tumor vasculature. After DMXAA injection, tumor endothelial cells experience G2/M cell cycle arrest (21), rapidly apoptose and rupture, leading to increased tumor permeability (22–24). DMXAA-induced cytokine secretion from macrophages and DCs (25) play a key role in immune activation and tumor vessel damage. Specifically, serotonin, nitric oxide, the C-X-C motif chemokine ligand 10 (CXCL10 also called interferon-γ-inducible protein 10 (IP-10)), tumor necrosis factor (TNFα) and interferon-beta (IFNβ) (15, 26, 27) can all modulate endothelial cells and the immune system. These properties make STING agonists an attractive therapeutic option that could be used for mesothelioma, therefore we used DMXAA for proof-of-concept studies aiming to assist with treatment protocol design when using STING agonists in human mesothelioma.

We have shown that chemotherapy-induced tumor cell death expands the T cell response to dominant and subdominant epitopes using murine AE17sOVA that expresses ovalbumin (OVA) as a neo tumor antigen in which SIINFEKL is the dominant MHC class I epitope of OVA, whilst KVVRFDK is a subdominant epitope (28). Revealing weaker tumor antigens to the immune system could benefit the host response (29–31), and this is most likely to occur if DMXAA-induced tumor cell death elevates tumor antigen presentation. To our knowledge, no other studies have addressed the effect of a STING agonist such as DMXAA on hierarchical anti-tumor cytotoxic T lymphocyte (CTL) responses.

This study aimed to identify a DMXAA treatment regimen that: modulates mesothelioma tumor vessels; enhances tumor antigen presentation and promotes functional dominant and subdominant tumor antigen-specific CTL that infiltrate mesothelioma to mediate tumor regression. We further hypothesized that DMXAA could render the tumor microenvironment more permissive and responsive to biological molecules such as agonist anti-CD40 antibody or IL-2 to further enhance the immune response.

## 2 Materials and methods

### 2.1 Vadimezan, 5,6-dimethylxanthenone-4-acetic acid (DMXAA)

DMXAA, kindly provided by Professor Lai Ming Ching (Auckland Cancer Society Research Centre, New Zealand) as the sodium salt, was freshly dissolved in PBS for each experiment.

### 2.2 Tumor cell lines

AE17 and AB1 are murine mesothelioma cell lines derived from the peritoneal cavities of C57BL/6J and Balb/c mice respectively after injection with asbestos fibres (32). Injection of AE17 cells into naïve mice results in mesothelioma tumors histologically similar to human mesothelioma (32). AE17sOVA was developed by transfecting the AE17 parental cell line with cDNA for secretory ovalbumin (AE17 sOVA) so that in AE17-sOVA tumor-bearing C57BL/6J mice, OVA becomes a spy neo tumor antigen (32). Tumor cells lines were maintained in media consisting of RPMI 1640 (Invitrogen) supplemented with 10% fetal calf serum (FCS; Invitrogen, Auckland, New Zealand), 50μg/ml gentamicin (Pharmacia, Bentley, Australia) and 60μg/ml benzylpenicillin (CSL, Melbourne, Australia). Transfected tumor cell lines were maintained in the same medium supplemented with 400μg/L neomycin analog G418 (Geneticin; Invitrogen). Cells were cultured at 37°C in a 5% CO_2_ humidified atmosphere.

### 2.3 MTT assays

MTT (3-(4,5-dimethylthiazol-2-yl)-2,5-diphenyltetrazolium bromide) tetrazolium reduction assays assess cellular metabolic activity due to the enzymatic action of mitochondrial dehydrogenase present in viable cells providing an indicator of cell viability and proliferation. Cells were seeded into 96 well plates (Becton Dickinson, USA) at 5 x 10^3^ cells/well for 24 hours at 37˚C in 5% CO_2_. DMXAA was diluted 1mg/ml and 50µl added to 150µl RPMI for serial dilution in RPMI without serum; 100µl of DMXAA was added to each well in duplicate for 24-hour incubation. Each well was treated with 50µl of 2mg/ml 3-(4,5-Dimethylthiazol-2-yl)-2,5-diphenyltetrazolium bromide in PBS and incubated for 4 hours at 37°C in the dark. After 5-minute centrifugation at 1000 rpm at 25˚C, cell pellets were treated with 100µl DMSO, and incubated in the dark for 30 min at RT with continuous shaking. Wells were read at 595nm using a Biorad 3550 microplate reader (Hercules, CA, USA).

### 2.4 Propidium iodide staining

PI integrates with DNA indicating the amount of DNA present. PI staining intensity is directly proportional to the amount of DNA present in a cell, representing the cell cycle status of the cell (the G1, S, G2, and M phases). Six-well plates seeded with 1.5 × 10^5^ cells/ml in media and incubated at 37°C and 5% CO_2_ for 24 hours were treated with different DMXAA concentrations and incubated under the same conditions for a further 24 hours. Adherent cells removed using trypsin were centrifuged at 400g for 10 minutes when the pellet was re-suspended in sample buffer (1g glucose in 1L PBS) and centrifuged at 400g for 10 minutes; this step was repeated once more. Cells were then vortexed in 70% ice-cold ethanol, fixed overnight at 4°C, vortexed and centrifuged at 3,500g for 5 minutes. The ethanol was discarded and the cell pellet vortexed to re-suspend cells in residual ethanol and 300ul PI staining solution (100mg PI, 100ml H_2_O; 21mg RNase A, 10ml sample buffer) added to each tube for 30 minutes in the dark at RT. Samples were analyzed using flow cytometry (BD CantoII flow cytometer, and Diva software for acquisition; FlowJo software for analysis).

### 2.5 Mice and ethics approval

Female C57BL/6J mice aged 6-8 weeks obtained from the Animal Resources Centre (Perth, Western Australia) were maintained under standard animal housing conditions at Curtin University’s animal holding facility. The OT-1 (H-2b) TCR transgenic mouse line, expressing a TCR recognizing the dominant H-2b OVA epitope (SIINFEKL) was kindly supplied by Professor I. Frazer and Dr. R. Steptoe (University of Queensland, Australia) and bred in house at the University of Western Australia (UWA) animal holding facility. All mice were used in accordance with institutional guidelines and UWA and Curtin University’s Animal Ethics Committee approval (AEC approval numbers: 2011_16; 2012_21; 2013-03; RA/3/300/37).

### 2.6 *In vivo* tumor growth

Eighty percent confluent AE17 and AB1 tumor cells were harvested using trypsin and prepared for injection by washing twice in PBS. Viability was always greater than 90%. Mice were injected subcutaneously (s.c.) in the left flank with 5 x 10^5^ tumor cells per mouse in 100 μl of PBS. Mice were monitored daily and tumors measured using microcallipers. Mice were sacrificed using methoxyflurane (Medical Developments International, USA) in accordance with specific AEC approval requirements.

### 2.7 Harvesting tumors and organs for FACS staining

After removing tumors and organs, single cell suspensions were obtained by gently mashing between two frosted glass slides, removing debris by filtration through 30μm nylon mesh (BD Falcon), centrifuging cells at 1200 rpm for 6 minutes and re-suspending the cell pellet in PBS/2% TBS. Where relevant, lysis buffer (1M Tris-HCL, pH 7.2) was used to lyse red blood cells.

### 2.8 FACS staining and analysis

Cells were washed in PBS/2% TBS, adjusted to 10^6^/ml and 200 μl/well transferred into 96 well plates (Falcon) for staining with 50μl of antibodies: CD3-PE (clone 145-2C11, hamster IgG1, Beckton Dickinson (BD)), CD8-PerCP-Cy5-5 (clone 53-6.7, rat IgG2a, BD) and CD4-APC-Cy7 (clone GK1.5, rat IgG2b BioLegend) or their isotype controls (BioLegend). Cells were incubated for one hour at 4°C, washed in PBS and fixed in 2% paraformaldehyde for 15 minutes at 4°C, all in the dark. Fixed cells were washed in PBS/2% TBS and data acquired on a FACSCanto II (BD) using Diva software and analyzed using FlowJo v10 software (BD).

### 2.9 *In vivo* analysis of tumor antigen cross-presentation

The AE17-sOVA model was used to assess the ability of antigen presenting cells (APCs) such as DCs to present the neo tumor antigen (OVA) *in-vivo* using adoptively transferred T cells from OT-I TCR transgenic mice, which express a TCR recognizing the MHC class I H-2^b^-restricted dominant OVA epitope, SIINFEKL (28, 33). OT-1 cells were labeled with 5,6-carboxy-succinimidyl-fluorescein-ester (CFSE; Molecular Probes, Eugene, OR), as described (7, 28, 32, 33). Briefly, lymph node (LN) and spleen cells from OT-1 mice were re-suspended in RPMI at 2 x 10^7^cells/ml and incubated with 2.5μM CFSE (stock solution 5mM in DMSO) for 10 min at RT. Cells were washed through an FCS underlay twice and PBS alone twice, and 10^7^ cells injected i.v. into each recipient mouse. CFSE-labeled cells were recovered from lymphoid organs 3 days post adoptive transfer and OT-1 CD8^+^ cells analyzed by FACS analysis.

### 2.10 Immunohistochemistry

Cryocut sections (10µm) fixed in fresh 4% paraformaldehyde for 20 minutes were washed in PBS and sequentially blocked with 1% hydrogen peroxide (H_2_O_2_) solution (6% stock, Orion Laboratories, Perth, WA, Australia), avidin blocking solution A, followed by biotin blocking solution B, for 10-15 minutes (Vector, Burlingame, CA, USA). After one PBS wash, sections were incubated at RT for 45 mins with a rat anti-mouse Platelet Endothelial Cell Adhesion Molecule-1 (PECAM or CD31) IgG2a antibody (clone 390; BD) or the rat IgG2 isotype control, washed three times followed by sequential incubations with a biotinylated anti-rat antibody (Jackson ImmunoResearch Europe Ltd, UK) for 45 minutes and streptavidin-horse-radish peroxidase (DAKO, Denmark) for 30 minutes. To detect color, Sigma FAST™ (D-4168) DAB (3,3’-Diaminobenzidine) tablets were used as a peroxidase substrate, as per manufacturer’s instructions. Slides were rinsed in PBS, counterstained with Mayer’s hematoxylin (Sigma, St. Louis, MO) for 2 mins, washed with tap water, mounted in aqueous mounting media (Shandon Immuno-mount, Pittsburg, PA) with cover slips (Esco cover glass, Biolab Scientific, Canada).

### 2.11 Peptides

The dominant peptide OVA_257-264_ (SIINFEKL) and subdominant peptide OVA_55-62_ (KVVRFDKL) were manufactured by the Centre for Cell and Molecular Biology (University of Western Australia, Perth) at a purity of >89%.

### 2.12 *In vivo* CTL assay

We used our modified ‘3 peak’ *in-vivo* CTL assay (28, 34, 35). Briefly, after RBC lysis pooled C57BL/6 spleen and lymph node cells were washed and divided into three populations. One population was pulsed with 10^-6^M SIINFEKL and a second population pulsed with 10^-6^M KVVRFDK for 90 min at 37°C, then washed in PBS and labeled with high (5µM) or low (0.05µM) CFSE concentrations respectively. Control uncoated target cells (the 3^rd^ population) were labeled with an intermediate concentration of CFSE (0.5µM). For i.v. injection, 1 x 10^7^ cells of each population were mixed in 200µl PBS per recipient mouse. Specific *in-vivo* cytotoxicity was determined by collecting lymphoid organs and tumors 18 hours later, and CFSE^+^ cells detected by flow cytometry.

The ratio between the percentages of uncoated versus peptide-coated cells (CFSE^Int^/CFSE^high^) was calculated to obtain a numerical value of cytotoxicity for each mouse. Further controls included naïve (no-tumor) mice, mice bearing parental AE17 tumors that do not express OVA and tumor-bearing PBS-treated recipient mice. To normalize data, allowing inter-experimental comparisons, the data was expressed relative to the no tumor control mice that were included in every experiment, as previously described (28, 35), i.e. relative killing was calculated by determining the ratios between the percentages of peptide-coated targets in no tumor control mice versus tumor-bearing mice and multiplying by 100 to obtain a percentage value.

### 2.13 Agonist anti-CD40 antibody and IL-2 treatment

Agonist rat anti-murine CD40 antibody (Ab) (FGK45) kindly provided by Professor C. Melief (Leiden University Medical Center, Netherlands) was expanded by the Monoclonal Antibody Facility (Perkins Institute, Perth, Western Australia) and diluted in PBS to 0.4mg/ml so that 40μg/100 μl/injection could be delivered intra-tumorally (i.t.) *via* a 26-gauge needle. Endotoxin levels were < 0.1 EU/ml (measured by the supplier). Lyophilized PROLEUKIN^®^ (aldesleukin, recombinant human IL-2; Clinigen Health Care, UK) was reconstituted in sterile PBS (Sigma) and routinely assayed for its bioactivity using a murine IL-2-dependent cytotoxic T cell line (CTLL), as previously described (32). Six i.t. injections per mouse of IL-2 (20µg/injection) or anti-CD40 antibody were given three times per week for two weeks, as previously described (32, 36).

### 2.14 Statistical analysis

Statistical analyses were performed using GraphPad Prism 8 (GraphPad Software Inc., California, USA). One-way Analysis of variance (ANOVA) was used to compare differences between multiple groups followed by a multiple comparisons test to compare two selected groups. The Mann-Whitney U test (unpaired, two-tailed) was used to examine differences between two groups; p values of less than 0.05 were considered significant.

## 3 Results

### 3.1 DMXAA can exert direct toxic effects on mesothelioma cells

**Figure 1:**
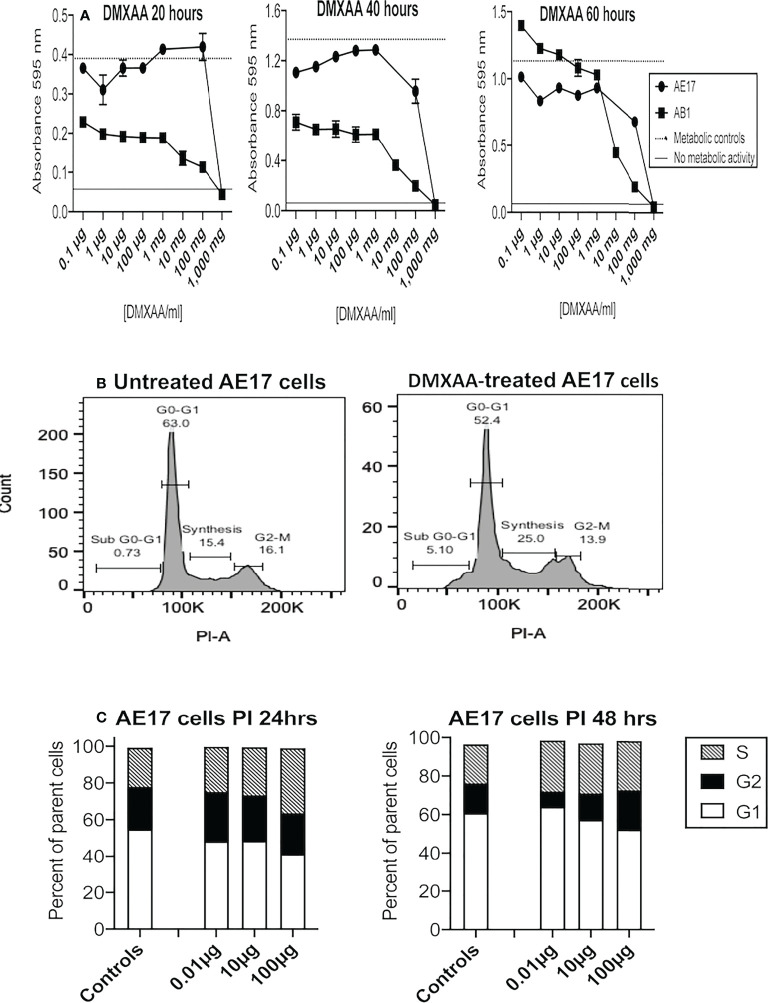
Low DMXAA doses modulate cell cycle, high doses induce metabolic dysfunction in mesothelioma cells. AE17 and AB1 mesothelioma tumor cells were treated with varying concentrations of DMXAA for 20, 40 and 60 hours and metabolic dysfunction assessed using MTT assays **(A)**. Data shown is from one of three experiments, each using duplicates and all demonstrating a similar trend. AE17 cells treated with three low doses of DMXAA for 24 and 48 hours were stained with PI and analyzed by flow cytometry. Representative histograms of untreated and DMXAA-treated AE17 cells **(B)** show the G1, S and G2 phases of the cell cycle. Pooled data from two experiments each with duplicates for DMXAA-treated AE17 cells and 5 replicates for untreated controls **(C)** shown as mean ± SEM; differences between controls and each of the DMXA doses for G1 versus G2 and G1 versus S were statistically significant at 24 and 48 hours (p < 0.005).

The first experiments examined whether DMXAA exerted toxic effects on two murine mesothelioma cell lines (AE17 and AB1) using the MTT assay. At 20 and 40 hours the MTT data suggest low doses between 0.1 and 100 µg/ml increased metabolic activity, and by 60 hours metabolic activity was higher than the controls at the same doses (**Figure 1A**). However, at higher DMXAA concentrations (> 1 mg/ml) AE17 and AB1 mesothelioma cell metabolic dysfunction dramatically increased.

Flavones such as DMXAA exert their cytotoxic effect by interrupting the cell cycle at the G2/M phase (37). Therefore, we assessed the effects of DMXAA on AE17 cell cycle. DMXAA concentrations up to 100 µg/ml pushed AE17 cell cycle into the DNA synthesis (S) phase at 24 hours and then into the G2 phase at 48 hours, suggesting low DMXAA doses could stimulate mitosis in mesothelioma cells (**Figures 1B, C**) as shown in **Figure 1A**. These data show that high concentrations of DMXAA exert direct toxic effects on mesothelioma cells, whilst lower doses might induce proliferation.

### 3.2 Modulating DMXAA dosage/regimen/route is critical for safe effective anti-mesothelioma activity

DMXAA has been reported to induce tumor regression following intravenous (i.v.) (often used in human clinical trials such as (38, 39), intraperitoneal (i.p.) (12, 16, 18) and intratumoral (i.t.) (40) delivery.

Therefore, we aimed to identify an effective dose and treatment regimen to treat murine mesothelioma. Based on our previous studies using immunotherapy, we hypothesized that tumor burden may influence DMXAA dose and/or treatment regimens. We found that small tumor burdens of less than 25mm^2^ are highly susceptible to i.t. IL-2 or anti-CD40 monotherapies (7, 32). We propose that these small tumors represent ‘early stage tumors’; they weigh 0.15 to 0.3g (representing ~ 1% of total body) and are well enough established to contain their own blood supply. However, we found that single-agent immunotherapies failed at a precisely defined ‘cut-off’ tumor burden; i.e. 25 mm^2^. Therefore, we defined AE17 tumors < 25mm^2^ as small, and those > 25mm^2^ as large, with the latter more resistant to immunotherapy. We have also shown that as AE17 mesothelioma tumors develop in size, tumor macrophage-associated macrophages adopt a more suppressive phenotype and transition from M1-like anti-tumorigenic macrophages to M2-like pro-tumorigenic macrophages (we termed M3 macrophages) (refs). We believe that larger tumors better represent human mesothelioma, as they are mostly associated with a longer time to develop. Therefore, we propose that DMXAA needs to overcome the greater suppressive tumor microenvironment seen in larger tumors before it could be considered a potentially translatable therapeutic. We have also shown that local delivery of immunotherapy is more effective and less toxic than systemic delivery (7, 32). Therefore, the effectiveness of DMXAA was tested on small and large tumors given i.v., i.p. or i.t.

**Figure 2:**
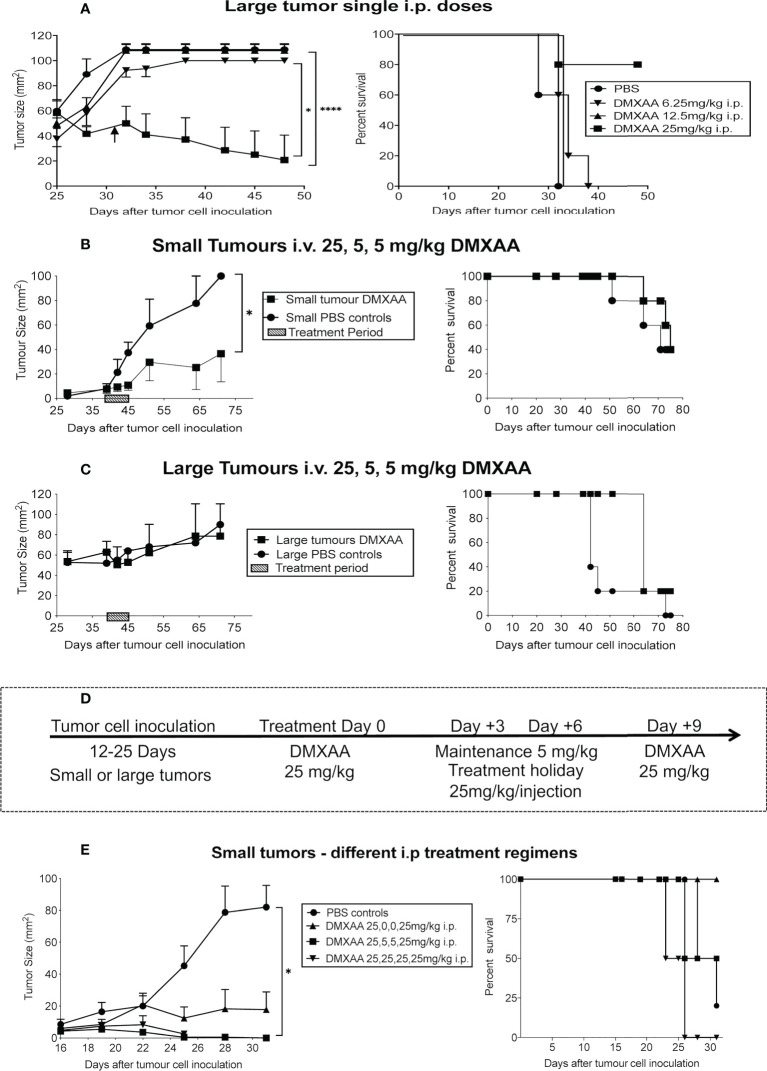
Fine-tuning DMXAA dosing and regimens. After tumor cell inoculation into C57BL/6J mice, AE17 tumors were left to develop into large tumors (≥40mm^2^) before one i.p. dose of DMXAA ranging from 6.25 mg/kg to 30 mg/kg (n = 5 mice/treatment group), with PBS controls **(A)**. In a separate experiment, AE17 tumor-bearing mice were divided into four groups (n = 3-4 mice/group); 2 groups with small **(B)** and 2 groups with large tumors **(C)**. All groups were given i.v. 25mg/kg DMXAA plus two 5mg/kg doses 3 days apart. Another group of mice with small tumors were divided into 4 groups (n = 5 mice/group). Group 1 had 25,5,5,25mg/kg DMXAA; Group 2 had 25,0,0,25mg/kg; Group 3 had 25,25,25,25mg/kg; Group 4 was PBS controls. Each group was given 4 i.p. injections 3 days apart **(D, E)**. Tumor growth (mean ± SEM) and Kaplan Meier survival plots shown. * p < 0.01; **** P < 0.0001.

Others have shown that the maximum tolerated dose (MTD) for DMXAA in mice is between 25mg/kg (22) or 30mg/kg (27). Therefore, we selected 30 mg/kg as our highest testable dose. Mice bearing large tumors (> 40 mm^2^) were treated i.p. with one dose ranging from 6.25mg/kg to 30mg/kg DMXAA. The lower doses demonstrated minimal anti-tumor or toxic effects (**Table 1** and **Figure 2A**). However, close monitoring showed that 30mg/kg DMXAA i.p. was toxic after the first injection. Similar toxic issues were seen in mice given 30mg/kg DMXAA i.t. after the second dose (**Table 1**). In contrast, mice given one dose of 25mg/kg i.p. demonstrated tumor regression without obvious toxic side effects, although no cures were seen (**Figure 2A**). Therefore, in our hands 25mg/kg DMXAA is the MTD.

**Table 1:** Summary of the treatment schedules and routes tested.

DMXAA dose/regimen	Route	Mesothelioma	
Single dose 6.25 mg/kg	i.p	*Large tumors*: No tumor effect (0%	No toxicity
Single dose 12.5 mg/kg	i.p	*Large tumors*: No tumor effect (0%	No toxicity
Single dose 25 mg/kg	i.p	*Large tumors*: Delayed tumor growth Relapsed after day 60	No toxicity
Single dose 30 mg/kg	i.p	Euthanised day 2	Toxic
2 doses 25,25 mg/kg 9-day intervals	i.p	*Small tumors:* Cured 1/4 (25%) Relapsed after day 30	Well tolerated
3 doses 25,25,25 mg/kg 7-day intervals	i.p	*Small tumors:* Delayed tumor growth 2/4 (50%) Relapsed after day 30	Well tolerated
4 doses 25,5,5,25 mg/kg 3-day intervals	i.p	*Small tumors:* Cured 2/4 (50%) Relapsed after day 28	Toxic
4 doses 25,25,25,25 mg/kg 3-day intervals	i.p	*Small tumors:* Cured ¼ (25%) Relapsed after day 25	Toxic
3 doses 25,5,5 mg/kg 3-day intervals	i.v.	*Small tumors:* Cured 1/5 (20%) Relapsed after day 75 *Large tumors*: No tumor effect (0%)	Well tolerated
Single dose 30mg/kg	i.t.	Tumor reduction after 1 dose Euthanised day 3	Toxic
3 doses 25,25,25 mg/kg 9-day intervals	i.t.	*Small tumors*: Cured 5/5 (100%) Relapsed after day 72 *Large tumors*: Cured 3/4 (75%) Relapsed after day 72	Well tolerated

The light grey rows show treatment schedules ruled out due to evidence of toxicity and/or minimal tumor responses regardless of tumor size. The darker grey rows show treatment schedules ruled out due to a minimal response in large tumors.

A second experiment consisted of four groups of AE17-tumor-bearing mice. Two groups commenced treatment when their tumors were small, the other two groups had large tumors. The DMXAA dose and treatment regimen for all groups was 25mg/kg, followed by two maintenance doses of 5mg/kg, with 3-day intervals given i.v. Tumor reduction was seen in small, but not large, tumors (**Figures 2B, C**).

Another group of 20 mice with small tumors were given different i.p. DMXAA regimens all starting with 25mg/kg. Each group was given 4 injections 3 days apart (**Figure 2D, E**). Group 1 had 25,5,5,25mg/kg, with the 5mg/kg seen as maintenance doses; Group 2 had 25,0,0,25mg/kg, with two treatment holidays meaning the two 25mg/kg doses were given 9 days apart; Group 3 had 25,25,25,25mg/kg; Group 4 were controls given 4 PBS injections. In Group 1 2/4 mice were cured, with 2/4 mice showing long-term survival, however toxicity issues were observed. A better, less toxic response was seen in Group 2 with all 4 mice cured and demonstrating longer-term survival. In contrast, in Group 3 mice given the higher dose every 3 days only 1 was cured, but none survived long-term, possibly due to associated toxicity issues (**Figure 2D**).

Other regimens were also tried. For example, a 3-dose regimen consisting of 25mg/kg for the first treatment followed by 2 weekly (rather than 3 days) maintenance doses of 5mg/kg, all given i.p. induced short-term tumor regression in 2/4 mice (summarized in **Table 1**).

**Figure 3:**
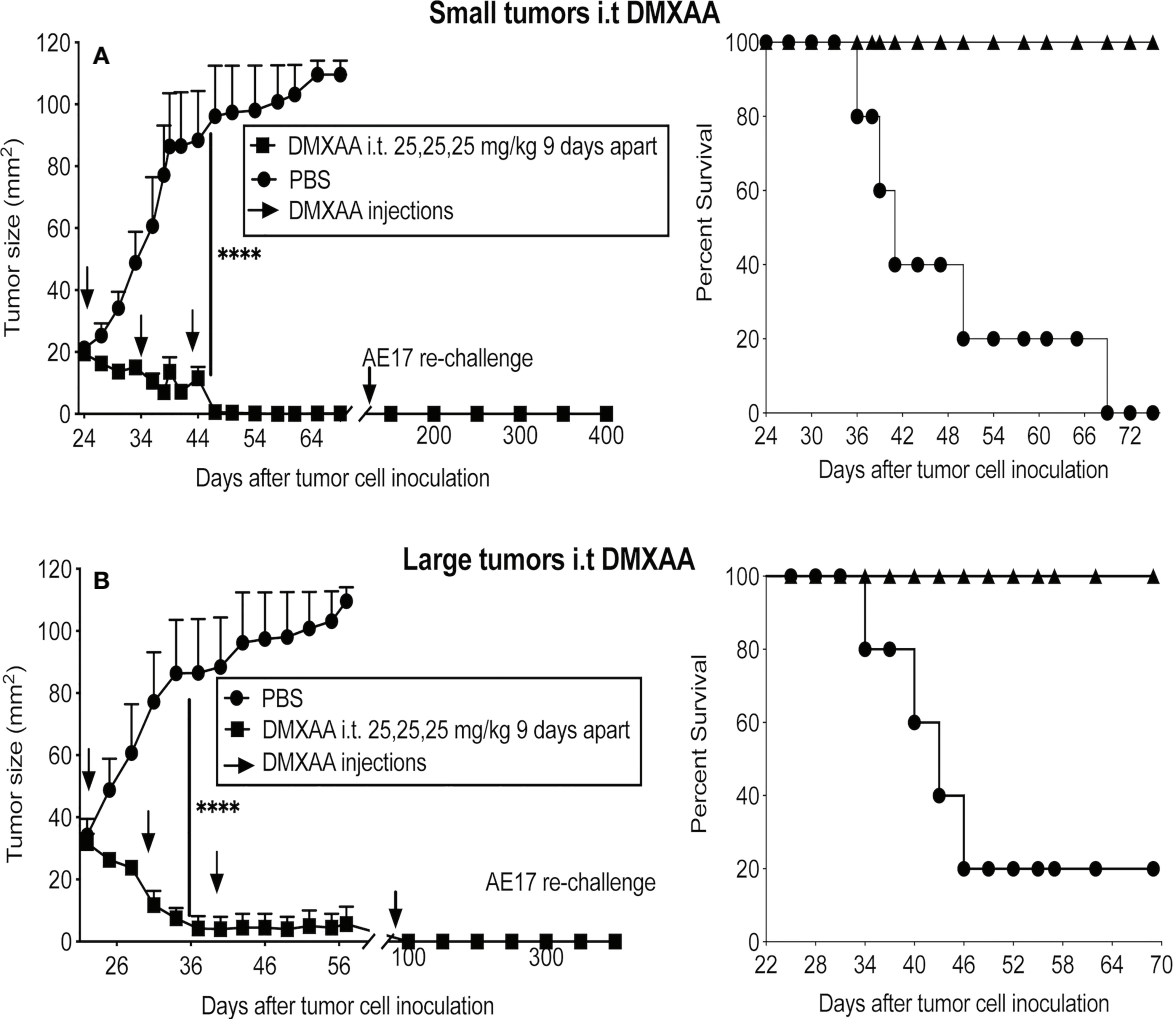
DMXAA exerts reliable anti-tumor effects upon intra-tumoral delivery. AE17 tumors were left to develop into small **(A)** and large **(B)** tumors before being given three i.t. doses of 25 mg/kg DMXAA with 9-day intervals (n = 5 mice/group), **** P < 0.0001.

Three doses of 25mg/kg DMXAA at 9-day intervals demonstrated the most significant reduction in tumor burden when given i.p. Therefore, this regimen was tested using the i.t. route in small and large tumors. All tumors in both groups resolved, leading to long-term survival (**Figures 3A, B**). Mice that were tumor free after 4 months were re-challenged with AE17 cells; no tumors emerged suggesting induction of protective memory immunity (**Figures 3A, B**).

**Table 1** summarizes the treatment schedules and routes tested. The light gray rows in **Table 1** show treatment schedules ruled out due to evidence of toxicity and/or minimal tumor responses regardless of tumor size. The darker gray rows show treatment schedules ruled out due to a minimal response in large tumors, as mesothelioma is a silent disease that is often diagnosed at a late stage. Taken together, the data showed that multiple doses given i.p. with at least a 7-day interval provided the safest and most effective outcomes. We tested the i.t. route and hypothesized that local delivery would be less toxic than systemic delivery, however a single dose of 30mg/kg given i.t. was toxic. In contrast, the i.t. route with a 9-day gap between three 25mg/kg doses proved the most effective schedule in small and large tumors.

Overall, while the anti-mesothelioma effect of DMXAA given i.t. proved to be long-term it was not permanent, as between 5 to 8 months later > 60% of tumors re-emerged. The data show that careful dosing and regimen testing is required to avoid toxic side effects and induce an anti-tumor response, with the i.t. route being the most promising.

### 3.3 DMXAA enhances tumor antigen presentation

**Figure 4:**
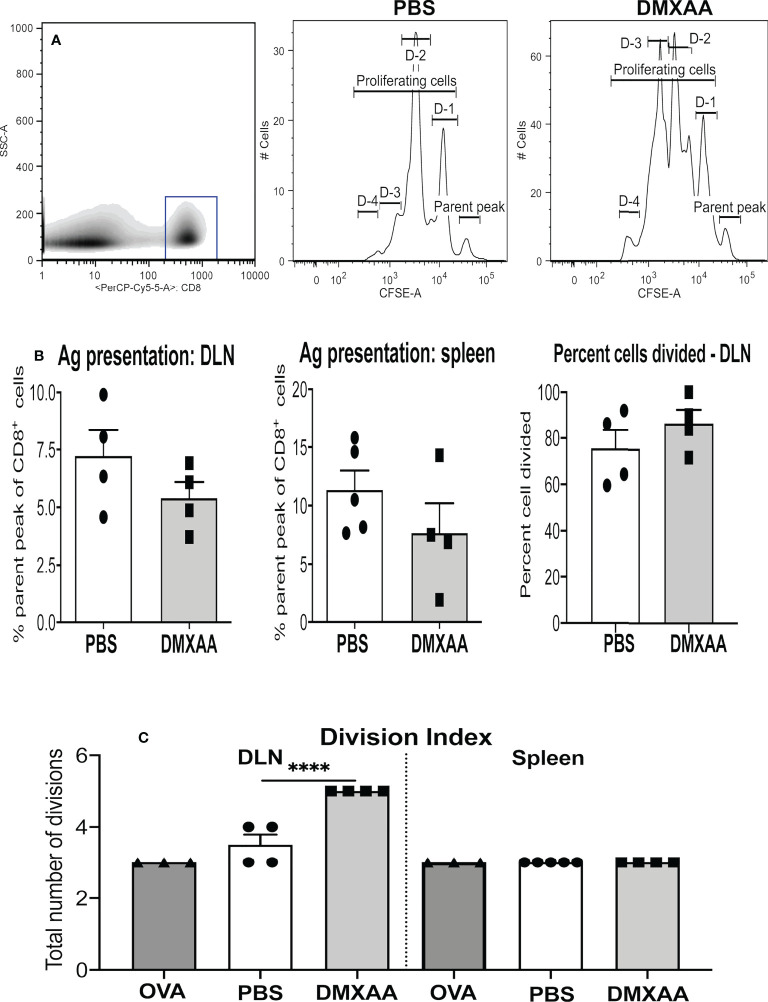
DMXAA promotes tumor antigen presentation to T cells. AE17sOVA-bearing mice were given two i.t. doses of PBS, or 25mg/kg DMXAA, or OVA (the positive control). Three days prior to analysis 1 x 10^7^ CFSE-labelled, tumor antigen-specific OT-1 CD8^+^ T cells were adoptively-transferred into all mice. Twenty-four hours later draining LN (DLN) and spleens were disaggregated into single cell suspensions, and stained for CD8 expression. Analysis of proliferating OT-1 cells was achieved by gating on CD8^+^ cells (shown in a representative density plot, A). Histograms of CD8^+^CFSE^+^ OT-1 T cells from a PBS-treated control and a DMXAA-treated mouse **(A)** show cells in the parent peak that have not divided, whilst cells in D-1 to D-4 peaks represent proliferating daughter cell that have undergone 1 to 4 divisions. Pooled data show symbols representing individual mice with columns indicating mean ± SEM for the percent of cells remaining in the parental peak in DLN and spleens, and the percent of cells that have divided in DLNs **(B)**. The number of divisions responding cells underwent is shown as a division index **(C)**. Data is from one experiment with PBS controls (n = 5), DMXAA-treated (n = 4), plus mice given the intact OVA protein (n = 2); **** = p < 0.0001.

To assess the impact DMXAA has on tumor-specific immunity mice were injected s.c. with AE17sOVA cells in which OVA becomes a tumor antigen. To avoid tumor antigen loss complications, treatment commenced when tumors were small (16 mm^2^ to 25 mm^2^) with mice given two thirds of the effective dose to ensure sufficient tumor remained for analysis, i.e. AE17sOVA-bearing mice were given two i.t. injections of PBS or 25mg/kg DMXAA 9 days apart and left for four days before adoptive transfer of CFSE-labeled OT-1 cells, followed by organ collection 24 hours later. Proliferation of CFSE-labeled, SIINFEKL-specific CD8^+^ T cells was used as a real-time, *in-vivo* indicator of antigen presentation. Disaggregated cells from draining lymph nodes (DLN), non-draining LN, spleen, and tumors were stained for, and gated on, CD8^+^ cells and proliferating CD8^+^ OT-1 cells identified by CFSE expression, as previously described (28, 32, 41) (**Figure 4A**). DMXAA-treated mice were compared to PBS-treated controls. Reduction of the parental peak (i.e. OT-1 cells that have not proliferated) illustrates the quality of antigen presentation (**Figure 4A**).

In agreement with our previous studies, antigen presentation occurred naturally in the DLN and spleens of PBS-treated AE17sOVA-bearing mice (**Figures 4A, B**) (32). OT-1 T cell proliferative responses in mice given DMXAA trended towards a reduced parental peak (implying greater proliferation), however the differences were statistically insignificant (**Figures 4A, B**). Similarly, the percentage of cells dividing remained the same (**Figure 4B**), yet the division index significantly increased in DMXAA-treated mice relative to PBS-treated control mice (**Figure 4C**). The data show that DMXAA induces daughter T cells to undergo more divisions than those in PBS-treated control mice.

### 3.4 DMXAA is associated with blunted lytic function of tumor-specific CTLs

The data above suggested improved presentation of the dominant epitope of OVA, SIINFEKL. However, OVA expresses other epitopes that bind MHC class I H-2K^b^ molecules expressed by mice on the C57BL/6J background. OVA_55-62_ KVVRFDKL is classified as subdominant due to its capacity to induce weak CTL responses (33). Therefore, we examined CTL responses generated to the dominant and subdominant epitopes in AE17sOVA-bearing mice treated with or without two doses 25mg/kg of DMXAA 9 days apart.

**Figure 5:**
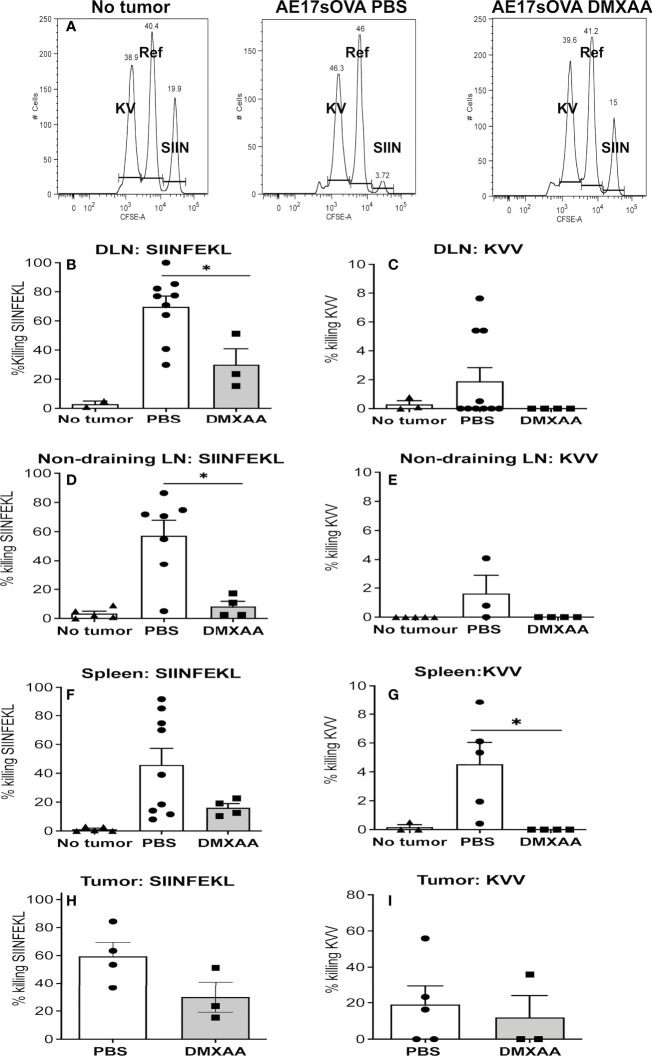
The quality of tumor-specific CTLs is blunted with DMXAA treatment. AE17sOVA-bearing mice were treated with two doses of PBS or 25mg/kg DMXAA. A further control group did not have tumors. Four days after the last dose, all mice were i.v. injected with target cells for an *in-vivo* CTL assay consisting of LN and spleen cells from naïve C57BL/6J mice divided into three populations. Eighteen hours later tissues were disaggregated into single cells for FACS analysis. Representative histograms are shown for no tumor controls, PBS-treated and DMXAA-treated mice with the SIINFEKL (SIIN)-pulsed population expressing high CFSE levels; the KVVRFDKL (KV)-pulsed population expressing low CFSE levels; and the no-peptide control target cells (Ref) expressing an intermediate level of CFSE **(A)**. Loss of target cells relative to controls indicates CTL killing. Data expressed as the percentage of SIINFEKL **(B, D, F, H)** and KVVRFDL **(C, E, G, I)** specific lysis, are shown in DLN **(B, C)** non-draining LN **(D, E)**, spleens **(F, G)** and tumors **(H, I)**. Symbols represent individual mice with columns representing the mean ± SEM of pooled data from 3 repeat experiments, each with 2 - 5 mice per group and total number per treatment group ranging from 2 to 9 mice; * p < 0.01.

The *in-vivo* CTL assay involves target cells carrying peptides on surface MHC class I molecules that can only be recognized by CTLs specific to each peptide. Thus, their disappearance due to killing by CTLs can be seen when analyzed by flow cytometry, as described (28, 35). In this assay, pooled target cells taken from the LNs and spleens of naïve C57BL/6J mice were divided into three populations. One population was pulsed with SIINFEKL and identified by labeling with a high concentration of CFSE *in vitro* (shown as SIIN in **Figure 5A**). A second population was pulsed with the subdominant peptide (KVVRFDKL) and labeled with a low CFSE concentration (shown as KV in **Figure 5A**). No peptide control target cells were labeled with an intermediate concentration of CFSE (shown as Ref in **Figure 5A**). The three populations were pooled and injected i.v. into mice four days after the last dose of DMXAA. FACS analysis of disaggregated organs was conducted 18 hours later.

The ratio between the percentages of uncoated (the Ref peak) versus peptide-coated targets (the KV or SIIN peaks, all shown in **Figure 5A**) (CFSE^ref^/CFSE^KV or SIIN^) was calculated to obtain a numerical value of cytotoxicity for each mouse. To normalize data, allowing inter-experimental comparisons, the data was expressed relative to the no tumor control mice that were included in every experiment. That is, relative killing was calculated by determining the ratios between the percentages of peptide-coated targets in no tumor control mice versus tumor-bearing mice and multiplying by 100 to obtain a percentage value.

We have shown a strong CTL response to the dominant epitope (SIINFEKL) in PBS-treated mice, with lower response levels to the subdominant (KVVRFDKL) epitope in DLNs, spleens, non-draining LN and tumor (28, 35) and **Figures 5B–I**). DMXAA-treated mice demonstrated a weaker SIINFEKL-specific CTL response relative to PBS-treated controls (**Figure 5B**). Unlike chemotherapy (28) DMXAA did not augment CTL responses to the subdominant epitope in any tissue, instead DMXAA reduced responses (**Figure 5B**). The data suggests that DMXAA blunts tumor-specific CTL activity.

### 3.5 DMXAA promotes infiltration of tumor-specific T cells

**Figure 6:**
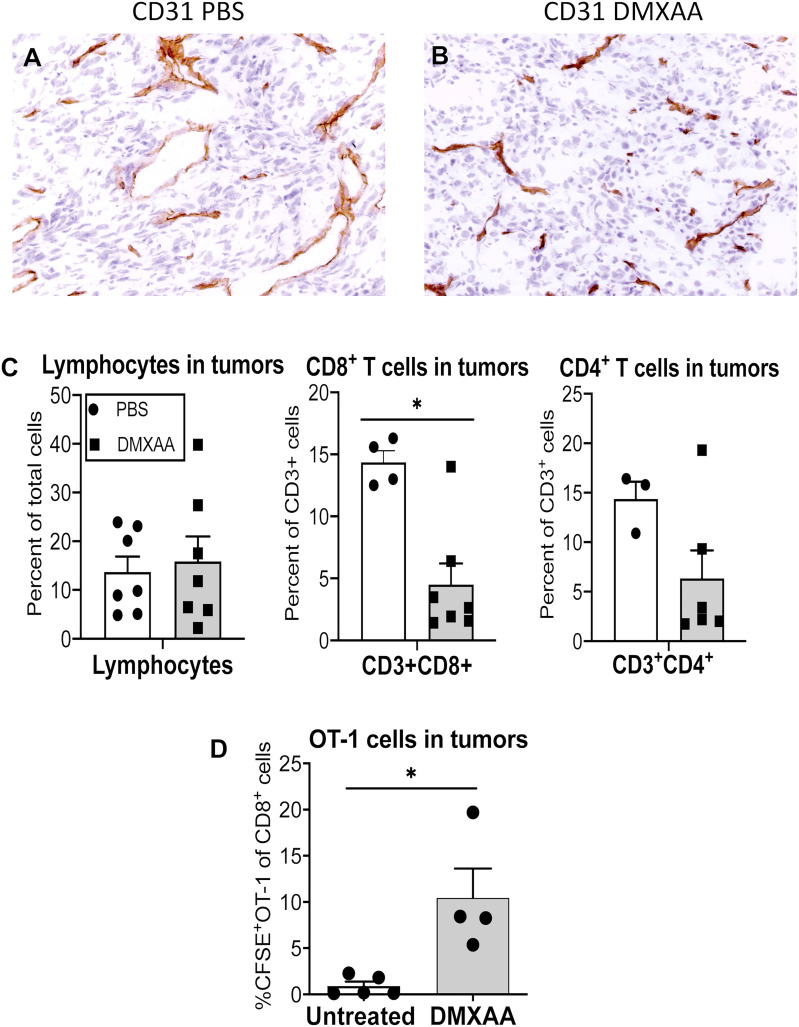
DMXAA increases the proportion of tumor-specific cell in tumors. Tumors from mice in the antigen presentation assay that received CFSE-labeled CD8^+^ OT-1 T cells (**Figure 4**) were preserved as frozen samples or freshly disaggregated into single cells. Immunohistochemical staining on frozen sections examined CD31 (PECAM)^+^ blood vessels in PBS **(A)** and DMXAA-treated **(B)** tumors. FACs analysis on single cells looked at CD3^+^CD8^+^ and CD3^+^CD4^+^ endogenous T cells, and at CD3^+^CD8^+^CFSE^+^ OT-1 cells. Tumor-infiltrating lymphocytes are shown as a percentage of total cells **(C)**. CD3^+^CD4^+^ and CD3^+^CD8^+^ T cells in tumors are shown as a percentage of CD3^+^ cells **(C)**. OT-I cells in tumors are shown as a percentage of CD8^+^ cells **(D)**. Symbols represent individual mice, columns represent pooled mean ± SEM for each group from 2 repeat experiments with a total number of mice ranging from n = 3 – 5 mice/treatment group; * p < 0.01.

Immunohistochemistry showed that DMXAA modulates CD31^+^ tumor vessels (**Figure 6A, B**) prompting us to ask if tumor-infiltrating lymphocytes changed. DMXAA did not change the proportion of lymphocytes within tumors (**Figure 6C**). In contrast, intra-tumoral CD3^+^CD4^+^ and CD3^+^CD8^+^ T cell proportions decreased with DMXAA-treatment relative to PBS controls (**Figure 6C**). The proportion of CFSE-labeled OT-1 cells that infiltrated tumors from the antigen presentation experiments was also examined. Very few OT-1 T cells were seen in tumors from PBS control mice. However, tumors from mice treated with DMXAA contained significantly higher levels of OT-1 cells (**Figure 6D**).

### 3.6 DMXAA should not be combined with an agonist anti-CD40 Ab or interleukin-2

**Figure 7:**
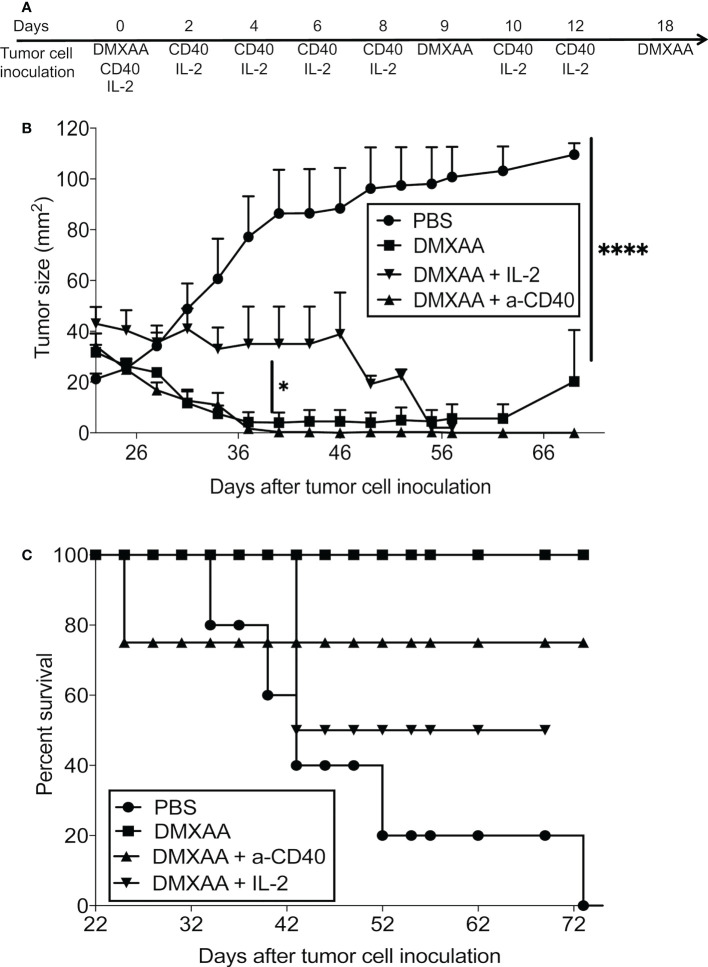
Combination with agents that induce CD40 or lL-2 signaling inhibits DMXAA efficacy. AE17-bearing mice (n = 4 - 5 mice per treatment group) were given 3 i.t. doses of 25 mg/kg DMXAA with 9-day intervals with or without 6 i.t. doses of anti-CD40 antibody 40μg/dose or 20µg IL-2/dose over 2 weeks (i.e. 3 doses/week for 2 weeks) **(A)**. Tumor growth **(B)** and survival **(C)** measured; * p < 0.01, **** = p < 0.0001.

We showed that DMXAA improved antigen presentation, yet CTL function was compromised, therefore we hypothesized that combining DMXAA with an agonist anti-CD40 antibody that activates DCs (7) or with IL-2 that expands tumor-specific T cells (32) might improve DMXAA efficacy. Therefore, we assessed the potential of combining DMXAA with the agonist anti-CD40 antibody, FGK45, or IL-2. Whilst combining DMXAA with IL-2 or anti-CD40 antibody reduced tumor size, long-term survival was compromised as mice that were apparently cured of their tumors died. More than 20% of mice treated with DMXAA plus anti-CD40 antibody did not survive beyond 23 days, whilst 50% of mice treated with DMXAA plus anti-CD40 antibody did not survive beyond 42 days. In contrast, all DMXAA only treated mice survived beyond 72 days. We speculated that toxicity was the cause of death for the combination therapies, but this was not investigated any further and the mechanisms causing death are unknown (**Figures 7A–C**).

## 4 Discussion

There is renewed interest in STING agonism for cancer treatment due to a deeper understanding of the mechanisms underlying the STING pathway and its indispensable role in innate and adaptive immunity. Moreover, STING agonists are being developed for use in humans. DMXAA remains a useful STING agonist for proof-of-concept studies in mice, and whilst DMXAA has been tested in another murine mesothelioma model syngeneic to Balb/c mice, i.e. AB12 (12), several questions remained unanswered for mesothelioma including: identifying an optimal treatment regimen; providing a deeper understanding of changes to tumor antigen presentation *in-vivo*; measuring changes to CTL hierarchical responses; and addressing the potential for combination with other immunotherapies.

### 4.1 Novel aspects of our study

We used our well-described murine mesothelioma model (32) to identify an optimal treatment regimen. Whilst this is a transplantable model, its strengths include being generated by the appropriate carcinogen, asbestos, in the peritoneal cavity of adult mice aged 8-12 weeks. The peritoneal cavity is lined with mesothelial cells and tumors of mesothelial cell origins developed >11 months after asbestos exposure; this slow development is similar to the long latency period seen in humans. Therefore, excised tumor cells from elderly asbestos-exposed mice were likely ‘shaped’ by the immune system before cloning, and better resemble human disease. Moreover, histopathology shows human mesothelioma-like features even when tumors are excised from a subcutaneous site (32). We are focusing on mesothelioma, and not aiming to provide a more broadly applicable therapeutic regimen.

Our first aim was to identify a long-term curative treatment protocol for mesothelioma using DMXAA. Others have shown that a single intra-peritoneal injection of DMXAA can induce tumor regression (12, 16, 18, 22, 42). Those papers mostly do not follow long-term outcomes; e.g. it does not look like cures were seen in the MMTV-PyMT breast cancer model (18), and the study goes out to 13 days after treatment. The minimum time point we go to is 38 days, with most of our time points being > 50 days.

Another novel aspect is that we found the treatment protocol that reliably induced long-term cures was i.t. delivery consisting of three injections given 9 days apart. This means that 25mg/kg DMXAA was directly delivered into the tumor bed. In contrast, intra-peritoneal injections represent a systemic treatment approach and it is unclear how much DMXAA localizes to tumors and how much disseminates around the body. Different local concentrations and systemic effects of DMXAA could induce different mechanisms, discussed below.

Our immunological analyses were performed at much later time points to others who closely examined very early responses starting 2-3 hours after treatment and ceasing approximately 7 days later (12, 16, 18, 22, 42). We examined immune responses 4-5 days after the second injection of DMXAA. The reason we chose that time point is that AE17 tumors lose expression of the marker tumor antigen (ovalbumin) not long after this time point (data not shown), which could confound the data. This antigenic loss is likely due to immune pressure, as functional lytic tumor-specific T cells are generated within the first week after tumor implantation, yet they do not prevent tumor progression, suggesting rapid acquisition of regulatory mechanisms that further sculpt tumors. This scenario is very likely reproduced in people with progressing mesothelioma. Mechanisms identified by others occurring between 2 hours and 7 days after a single i.p. injection could be very different to those operating after two i.t. injections given 9 days apart, discussed further below.

We addressed tumor specificity in CD8^+^ T cells and the ability of these T cells to recognize dominant and subdominant tumor antigens by looking at real-time T cell lytic function in tumors and lymphoid tissue using an *in vivo* CTL assay. To our knowledge this is completely novel. We hypothesized that increased tumor cell death induced directly and indirectly by DMXAA-induced effects on immune cells would expose tumor-associated DCs and T cells in LNs to a greater array of tumor antigens, including weaker subdominant antigens. This is because we had seen this phenomenon after chemotherapy-induced tumor cell death (28); the results are discussed below.

### 4.2 The optimal DMXAA treatment protocol for this mesothelioma model was intra-tumoral delivery with 3 repeated injections 9 days apart

We aimed to identify an optimal treatment regimen by testing a number of doses and treatment regimens. We found that DMXAA had a narrow therapeutic window. For example, one dose of 30mg/kg given i.v., two 30mg/kg doses given i.t., and four 25mk/kg doses given i.p. 3 days apart proved toxic. Introducing a lower maintenance dose of 5mg/kg between two 25mg/kg doses was promising, as 50% of tumors resolved, however, toxicity was still noted. The most effective i.p. treatment involved a treatment holiday, i.e. two 25mg/kg doses given 9 days apart. Tumors resolved in 100% mice and no significant toxicity issues were observed, but >80% tumors re-emerged after 5 months.

Our data suggests that targeting the tumor microenvironment is a safer and more effective approach, a concept we demonstrated using agonist anti-CD40 antibody with or without IL-2 (7, 32, 36). Targeting the tumor microenvironment with 3 doses of 25mg/kg DMXXAA 9 days apart *via* direct injection proved safe and effective, as 100% tumors regressed and all mice remained tumor free for at least 6 months. These data are in agreement with others utilizing i.t. delivery approaches. For example, direct delivery of a STING agonist into melanoma leads to tumor regression and potent systemic immunity (40). Similarly, injecting the STING agonist, cGAMP, intra-tumorally into murine melanoma and colon cancer led to control of injected and contralateral tumors (43). Furthermore, local delivery of a STING agonist has been shown to be effective with minimal toxicity in a TRAMP prostate cancer model (44). Others are developing i.t. approaches using nanotechnology for STING agonists (45, 46).

### 4.3 Intra-tumoral DMXAA reduces blood vessels and upregulates tumor antigen presentation yet blunts T cell effector function

We show that i.t. DMXAA up-regulated antigen presentation evidenced by adoptively transferred OT-1 T cells undergoing more divisions than those in untreated controls. These data suggest that DCs were activated whilst in the tumor before migrating to the DLN to engage tumor-specific T cells, and are in agreement with studies showing that DMXAA activates DCs (16).

Given the antigen presentation data, we were surprised to find a reduced proportion of CD4^+^ and CD8^+^ T cells in STING-activated tumors relative to PBS-treated tumors. Moreover, CTL lytic activity to the dominant and subdominant epitopes was compromised. Recent studies have shown high STING expression levels render T cells susceptible to STING activation, with strong signaling inducing apoptosis *via* elevated ER stress, unfolded protein response (UPR) gene expression and cell death pathways (17, 47–49) Doses > 5µg/ml DMXAA induce cell death in naive B6 T cells after 24 hours (17). We delivered 25mg/kg DMXAA directly into tumors, suggesting we are within toxic levels for T cells. Loss of CTLs could attenuate STING-driven anti-tumor benefits. These observations help account for data showing DMXAA treatment does not synergize with CD8^+^ T-cell immunotherapy (50). It is possible that the DMXAA dose we used provided a signal strength that enhanced the ability of DCs to present antigen but compromised CTL function. These data could also explain our findings using DMXAA combined with agonist anti-CD40 antibody or IL-2.

Conversely, we found an increase in tumor-specific OT-1 CD8^+^ T cells infiltrating STING-treated tumors. Similarly, the STING agonist, cyclic dinucleotide GMP-AMP (cGAMP) enhanced antitumor CD8^+^ T responses that controlled murine melanoma and colon tumors (43). It is difficult to reconcile these data with the above reports regarding T cells, although one possibility is that there is a proliferative threshold when regulatory mechanisms become activated. There is also evidence that the OT-1 allele can rescue CD8^+^ T cell death associated with chronic ER stress in mice (49, 51), although the authors were unsure of the mechanisms behind OT-1 TCR rescue.

### 4.4 Possible mechanisms occurring in AE17 mesothelioma after 2 DMXAA injections 9 days apart

Several studies have reported that a single i.p. injection of DMXAA is effective in slowing tumor growth and, in some cases, eliciting cures in different murine models, including AB12 mesothelioma (12), EG7 thymoma (16), colon-38 cancer (22), GL26 brain tumors (42) and the transplantable murine MMTV-PyMT breast cancer model (18). A single i.p. injection of 18-25mg/kg DMXAA in these models induced apoptosis in tumor vessels within 3 hours. An in-depth study in mice bearing PyMT breast cancer demonstrated rapid (in a few hours) damage to tumor vessels as well as waves of immune cell infiltration, starting with neutrophils, followed by monocytes and T cells (18). These authors showed that T cells enhanced the effector function of innate myeloid cells (18). Similarly, Wallace et al., showed that a single i.p. injection of 18mg/kg DMXAA led to cytokine release and tumor cell necrosis 24 hours later, and tumor reduction for up to 30 days, followed by relapse in EG7 tumors; activated DCs were seen by 24 hours in DLN followed by a rapid increase in the number of splenic tumor-specific CD8^+^ T cells (16). However, it was not clear from those studies if tumor-specific T cells had infiltrated tumors and whether they could lyse tumor cells *in situ*, although this was the expectation as T cells elevated IFN expression (18). We did not examine very early time points. Our data suggest that if this early DMXAA-induced effect occurred after the first i.t. injection, this response was not durable, as tumors returned. More injections were required to induce long-term tumor control, despite or because of blunted T cell responses; this is a novel finding.

The complexity of the mesothelioma microenvironment may contribute to the results. For example, we have shown that up to 50% of cells in AE17 mesotheliomas are macrophages, and that as AE17 tumors develop over time and in size, tumor-associated macrophages transition from M1-like anti-tumorigenic macrophages to M2-like pro-tumorigenic macrophages (termed M3 macrophages; 52). We do not know how mesothelioma-associated macrophages respond to DMXAA. This becomes complex because small tumors contain M1-like macrophages and large tumors contain M3 macrophages. Others have shown that DMXAA skews tumor associated M2 macrophages towards an M1 phenotype in models of non-small cell lung cancer (21) and AB12 mesothelioma (12); it is possible this is also happening in AE17 mesotheliomas. The remaining cells in the tumor microenvironment include tumor cells and other stromal cells, and it is difficult to understand how DMXAA affects each cell type and their consequent interactions. Moreover, the data using 30mg/kg demonstrated significant toxicity suggesting systemic leakage. Thus, whilst we delivered 25mg/kg into tumors we do not know how long this dose remained *in situ* and how tumor cells and other cells are affected. In our hands, high doses (> 1mg) of DMXAA directly induced AE17 tumor cell death; this was more effective over longer periods of time (40 and 60 hours). Lower doses (100µg or less) did not affect tumor cell viability and pushed cells into the G2/M phase of the cell cycle. We did not look at other cell types such as DCs, although others have shown that DMXAA activates DCs (16, 25). DMXAA-activated STING-expressing tumor-associated DCs could have emigrated to DLNs to induce OT-1 T cells to proliferate. Alternatively, STING-expressing resident DLN DCs exposed to leaked DMXAA could be responsible for OT-1 proliferation. Further studies are required to address these questions.

We have shown that three i.t. injections of IL-2 modified tumor blood vessels (32) and recruited CD8^+^ T cells into the tumor bed whilst three i.t. injections of IL-2 combined with an agonist anti-CD40 antibody recruited a massive and simultaneous infiltrate of neutrophils and T cells (7). Both immune cell types were required to permanently eradicate tumors, suggesting mutual signaling enhanced the effector function of both cell types. Similar findings were seen after a single intra-peritoneal injection of DMXAA into MMTV-PyMT breast bearing mice, i.e. T cells collaborated with myeloid cells to induce tumor regression (18). In our IL-2/CD40 studies, T cell numbers and lytic function were amplified and tumors were permanently eradicated. Therefore, we propose that one possible reason that we could not elicit permanent cures using DMXAA is that T cell lytic function becomes increasingly compromised *via* continuous STING signaling. Thus, we think DMXAA-driven anti-angiogenic effects, direct cytotoxic effects on mesothelioma cells and enhanced innate immunity play a key role in the tumor restraint seen in our system. However, without a sustained T cell response, mesothelioma tumors eventually escape immune attack and re-grow.

An un-answered question is how to best use this DMXAA-induced response in a combination setting so that long-term mesothelioma control is achieved without toxicity. One possibility is exploiting the potential of combining immune checkpoint inhibitors (ICIs) targeting the programmed death protein-1 (PD1) and cytotoxic T-lymphocyte antigen 4 (CTLA-4) pathways with a STING agonist. However, ICIs on their own or in combination, as first, second or third line treatment have yet to be fully evaluated before a consensus can be reached (53, 54; reviewed by 55–58) making it difficult for us draw conclusions regarding clinical combination with ICIs. Once a consensus has been reached, we will be better informed regarding moving forward with testing combining STING agonists and ICIs in the preclinical setting. We acknowledge that ICIs might not prove to be as effective as early clinical studies suggested (54, 59, 60; reviewed by 55, 56, 61, 62). Moreover, the loss of lytic function by T cells on account of local STING activation, and our inability to improve DMXAA outcomes by using factors that activate dendritic cells and T cells suggests combination with ICIs might not be effective. Further studies are required to address combination with ICIs.

In summary, we show that DMXAA enhanced mesothelioma-associated tumor antigen presentation and that tumor-specific T cells more readily infiltrated tumors. However, tumor-specific CTL function was blunted. Our data also shows that STING treatment regimens need to be carefully designed, as there is a fine line between achieving tumor resolution and maintaining immune control, particularly T cell control, without unwanted toxicity. Collectively, the data show that local delivery is the optimal approach that enhances tumor antigen presentation. However, there is a risk of compromising CTL function. Nonetheless, mesothelioma resolution was achieved, supporting further research involving the use of STING agonists for this devastating disease. The role of STING agonist in combination with contemporary clinically used checkpoint blockade targeting the PD1 and CTLA-4 pathways remains unclear.

## Data availability statement

The datasets generated during and/or analysed during the current study are available from the corresponding author on reasonable request.

## Ethics statement

The animal study was reviewed and approved by Curtin University Animal Ethics Committee and the University of Western Australia’s Animal Ethics Committee.

## Author contributions

DN and AN conceived the ideas and concepts, successfully applied for funding and oversaw all aspects of the project including data analysis and publication preparation. PG, SC and IL performed the experiments and analyzed the data. All authors contributed to the article and approved the submitted version.

## Funding

Funding from the Cancer Council of Western Australia and the New South Wales Workers’ Compensation Dust Diseases Board equally funded this project.

## Conflict of interest

Author IL was employed by Becton Dickinson Pty Limited.

The remaining authors declare that the research was conducted in the absence of any commercial or financial relationships that could be construed as a potential conflict of interest.

## Publisher’s note

All claims expressed in this article are solely those of the authors and do not necessarily represent those of their affiliated organizations, or those of the publisher, the editors and the reviewers. Any product that may be evaluated in this article, or claim that may be made by its manufacturer, is not guaranteed or endorsed by the publisher.
